# Evidence from a Mouse Model That Epithelial Cell Migration and Mesenchymal-Epithelial Transition Contribute to Rapid Restoration of Uterine Tissue Integrity during Menstruation

**DOI:** 10.1371/journal.pone.0086378

**Published:** 2014-01-22

**Authors:** Fiona L. Cousins, Alison Murray, Arantza Esnal, Douglas A. Gibson, Hilary O. D. Critchley, Philippa T. K. Saunders

**Affiliations:** Medical Research Council Centre for Reproductive Health, The University of Edinburgh, Edinburgh, Scotland, United Kingdom; AMS Biotechnology, United Kingdom

## Abstract

**Background:**

In women dynamic changes in uterine tissue architecture occur during each menstrual cycle. Menses, characterised by the shedding of the upper functional layer of the endometrium, is the culmination of a cascade of irreversible changes in tissue function including stromal decidualisation, inflammation and production of degradative enzymes. The molecular mechanisms that contribute to the rapid restoration of tissue homeostasis at time of menses are poorly understood.

**Methodology:**

A modified mouse model of menses was developed to focus on the events occurring within the uterine lining during endometrial shedding/repair. Decidualisation, vaginal bleeding, tissue architecture and cell proliferation were evaluated at 4, 8, 12, and 24 hours after progesterone (P4) withdrawal; mice received a single injection of bromodeoxyuridine (BrdU) 90 mins before culling. Expression of genes implicated in the regulation of mesenchymal to epithelial transition (MET) was determined using a RT2 PCR profiler array, qRTPCR and bioinformatic analysis.

**Principal Findings:**

Mice exhibited vaginal bleeding between 4 and 12 hours after P4 withdrawal, concomitant with detachment of the decidualised cell mass from the basal portion of the endometrial lining. Immunostaining for BrdU and pan cytokeratin revealed evidence of epithelial cell proliferation and migration. Cells that appeared to be in transition from a mesenchymal to an epithelial cell identity were identified within the stromal compartment. Analysis of mRNAs encoding genes expressed exclusively in the epithelial or stromal compartments, or implicated in MET, revealed dynamic changes in expression, consistent with a role for reprogramming of mesenchymal cells so that they could contribute to re-epithelialisation.

**Conclusions/Significance:**

These studies have provided novel insights into the cellular processes that contribute to re-epithelialisation post-menses implicating both epithelial cell migration and mesenchymal cell differentiation in restoration of an intact epithelial cell layer. These insights may inform development of new therapies to induce rapid healing in the endometrium and other tissues and offer hope to women who suffer from heavy menstrual bleeding.

## Introduction

The human endometrium displays a remarkable ability to undergo cyclical episodes of proliferation, angiogenesis, differentiation (decidualisation), inflammation and tissue breakdown (menses) occurring up to 400 times during a women’s reproductive life. Menstruation, the shedding of the upper functional layer of the endometrium, represents the culmination of a molecular cascade initiated by withdrawal of progesterone following the regression of the corpus luteum [1], [2]. Rapid restoration of tissue integrity at the time of menses is essential to avoid excess blood loss and to ensure the endometrium can regenerate in response to the sex steroid hormones oestrogen and progesterone in preparation for a potential pregnancy. The precise mechanisms responsible for repair of the endometrium, without scarring, are not fully understood. Recent microscopy studies have revealed that shedding of the endometrium is a locally occurring, progressive process, with areas of partially shed, as well as shed and regenerating endometrium observed in close proximity within the tissue [3], [4].

Re-epithelialisation, a crucial process in endometrial repair, occurs very rapidly and is independent of the actions of oestrogen [5]. Based on observations originally made by Novak and Te Linde, in 1924, it has been suggested that new populations of glandular and luminal epithelial cells arise from the epithelium of glands that are retained in the basal layer after shedding of the functional layer [6]. In the 1970s, Ferenczy suggested that the surface epithelium was derived from a simultaneous proliferation of cells at the exposed ends of basal glands and also from the persistent and intact surface lining that bordered the denuded areas of stromal tissue [5]. Recent data suggest that mechanisms contributing to endometrial repair may need to be revisited in light of results from studies on human endometrial stem cells [7], circulating progenitor cells [8], and human endometrial side population cells [9], all of which suggest novel role(s) in repair of the tissue following menses.

Our understanding of the mechanisms regulating menstruation has been informed by studies using human tissue explants and xenografts, the latter being maintained in mice with a reduced complement of immune cells [10], [11]. In a series of elegant studies, Marbaix and colleagues have demonstrated focal breakdown of matrix components within the stroma and highlighted the pivotal role played by matrix metalloproteinases [11]. Studies using macaques with artificially induced menstrual cycles, report increased expression of MMPs at menses, which complement studies in human tissues [12]. In mice, stromal cell decidualisation only occurs naturally in response to the presence of a blastocyst [13] and in the absence of a pregnancy the uterus is remodeled without shedding (menses). Finn and Pope were the first to describe a protocol for the use of hormonal injections and artificial induction of endometrial decidualisation in mice [14]. Stromal cell decidualisation was induced by oil injection and when progesterone support was withdrawn they exhibited features of menstruation including immune cell infiltration and tissue degeneration [14]. The model was later refined by Brasted et al [15] who used an inbred strain of mice to reduce intra-animal variation and altered the modes of delivery of the steroid hormones. They showed endometrial breakdown at 16 hours (the earliest time point) and complete endometrial regeneration by 48 hours [15]. This model has been widely used to complement and extend studies on human tissues documenting leukocyte infiltration at the onset of breakdown [16], changes in components of the extracellular matrix [17] and expression of metalloproteinases [18]. A recent study that used this model to examine the role played by prostaglandins reported administration of COX-2 inhibitors reduced endometrial breakdown and leukocyte recruitment [19] a finding that clearly recapitulates studies in women [20], [21].

The female reproductive system retains a remarkable degree of developmental plasticity that allows it to adapt to the challenges imposed by the menstrual cycle and pregnancy. Taylor and colleagues have highlighted the expression of members of the HOX gene cluster in the endometrium as evidence that the recapitulation of developmental processes is an essential feature of endometrial function [22]. Another process that plays a fundamental role in development, that may be recapitulated in adult tissues, are changes in cell behaviour so that they switch between mesenchymal and epithelial cell fates. This process is known as mesenchymal to epithelial transition (MET) with the alternative fate being defined as epithelial to mesenchymal transition (EMT) [23]. In this paper we report an updated and modified mouse model and highlight data that shed new light on the cellular and molecular mechanisms responsible for the rapid, immediate/early restoration of endometrial integrity.

## Materials and Methods

### Modified Mouse Model of Menstruation and Repair

All animal procedures were carried out in accordance with UK legal requirements and in under licensed approval from the UK Home Office. In the current study a mouse model of menstruation described by Brasted et al [15] was modified to include non-surgical induction of decidualisation and a longer decidualisation period. Uterine tissues were also collected during a period of active shedding and repair, time-points that have not been previously described.

**Figure 1:**
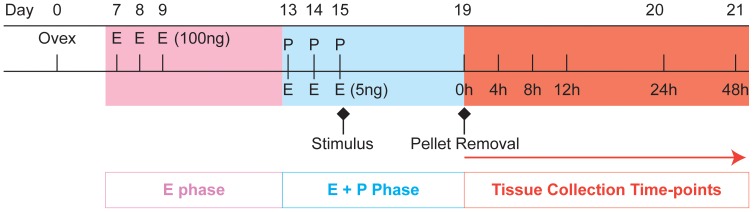
Summary of time line for mouse model of menstruation and regeneration. Colour coding pink = ‘proliferative phase’, blue = ‘secretory phase’, red = ‘menstrual phase’. Ovex; ovariectomy, E; β-oestradiol, P; progesterone. β-oestradiol concentrations in brackets (ng/100 µl), P4 pellet (1 mg/ml). One uterine horn was stimulated on day 15 via oil injection into the luminal cavity; “menses” was induced by P4 pellet removal on day 19. BrdU was injected 90 minutes prior to tissue recovery at 4, 8, 12 and 24 hours after the removal of the P4 secreting pellet.

On day 0, C57BL/6J mice between 8–10 weeks of age were ovariectomised to deplete endogenous steroid production. Mice received daily injections of β-oestradiol (E2) in sesame seed oil (100 ng/100 µl, days 7–9). A progesterone (P4)-secreting pellet was placed sub-cutaneously on day 13; mice also received daily injections of sub-cutaneous injections of E2 (5 ng/100 µl, days 13–15). On day 15, decidualisation of one uterine horn was induced by stimulation of the horn using sesame seed oil (20 µl) inserted into the uterine lumen via the cervix using a non-surgical embryo transfer device (NSET) from Datesand Ltd. (Manchester, UK). The contra-lateral horn acted as a control. P4 withdrawal was induced 90 hours after decidualisation by removing the P4-pellet. Mice were culled by asphyxiation and cervical dislocation at time of P4 withdrawal or 4, 8, 12, 24 and 48 hours thereafter (Figure 1). Mice received an intra-peritoneal injection of bromodeoxyuridine (BrdU, 2.5 mg/ml) 90 minutes prior to culling to detect cellular proliferation. Blood sera were collected, uteri dissected and collected into RNA later or 4% neutral buffered formalin. Any mouse in which the oil-treated horn had not decidualised was excluded from the study.

### Histology and Immunohistochemistry

All samples were fixed in NBF overnight at room temperature, rinsed in 70% ethanol and then stored in 70% ethanol, then processed into wax. Serial, transverse 5 µm sections were cut onto micro slides and incubated overnight. To determine gross morphology, sections were stained with haematoxylin and eosin. Single antibody immunohistochemistry [24] was carried out with antibodies directed against BrdU, WT1 and pan cytokeratin. Double immunofluorescence [25] was carried out with antibodies directed against vimentin and pan cytokeratin. Details of antibodies are provided in Table S1. Primary antibodies were diluted in serum (and incubated at 4°C overnight). Secondary antibodies were applied to sections for 30 minutes at room temperature (diluted 1/500), before the addition or a tertiary detection method, dependent upon the visualisation method used (immunohistochemistry (IHC) or immunofluorescence). In the case of IHC, streptavidin horseradish peroxidase was applied to slides for 30 minutes, positive signals were visualised using the chromogen 3,3-diaminobezidine (DAB). Reactions were stopped by immersion in water, sections were counterstained in haematoxylin. For immunofluorescence, slides were incubated with the tyramide signal amplification™ kit for 10 minutes, then with DAPI to enable nuclear counterstaining.

### RNA Extraction

Uterine samples were added to RNeasy Lysis Buffer (RLT) with 1% β-mercaptoethanol and homogenized. Lysates were added to RNeasy spin columns and total RNA was eluted according to manufacturer’s instructions (Qiagen). The total concentration of RNA in each sample was measured and then standardised to a concentration of 100 ng/µl using RNAse free water.

### Quantitative RTPCR

Reverse transcription of RNA to cDNA was performed using the Superscript VILO cDNA synthesis kit (InVitrogen) according to manufacturer’s instructions. Samples were incubated at: 25°C for 10 minutes, 42°C for 60 minutes and 52°C for 5 minutes in a thermal cycler. Primers for each gene of interest were designed using the Universal Probe Library Assay Design Center (Roche Applied Science) and purchased from Eurofins (MWG Operon), sequences are shown in Table S2. Reactions were prepared in duplicate. Amplification was carried out at 95°C for 10 minutes then 40 cycles of 95°C for 15 seconds and 60°C for 1 minute. Analysis was performed using the ΔΔCt method with the fold change calculated relative to an internal control (18 s) and to the 0 hour control sample set. Statistical analysis was performed using Graphpad Prism 5, significance was considered p<0.05 or less. Student t tests determined significance between the decidualised 0 hour time-point and the latter time-points.

### Array Analysis Using the RT2 Profiler PCR Array

RNA was analysed using Agilent Technologies according to the manufacturer’s protocol: only samples with a RIN>6.0 were used in the array. RNA samples (400 ng/µl) were treated to eliminate genomic DNA before preparation of cDNA. The RT2 profiler Mouse Epithelial to Mesenchymal Transition Array (SABiosciences, PAMM-090Z) was used with buffers supplied by the manufacturer. The full list of genes detected by the SYBR Green-optimized primer assays can be found at http://www.sabiosciences.com/rt_pcr_product/HTML/PAMM-090Z.html. A reaction mixture was prepared using the RT^2^ Real-Timer SyBR Green/ROX PCR Mix kit according to manufacturer’s instructions; amplification was carried out as described above. Analysis was performed using SABiosciences web portal, (http://pcrdataanalysis.sabiosciences.com/pcr/arrayanalysis.php), data analysis was performed using the ΔΔCt method comparing the 0 hour time-point to the 8 hour and 24 hour time-points as well as the 8 hour to 24 hour time-point.

### Progesterone ELISA

Determination of serum progesterone concentrations was carried out using a progesterone ELISA kit (DEMEDITEC diagnostics, Germany) as per manufacturer’s instructions. Samples were analysed in duplicate, standard curve and average absorbance values were calculated using Masterplex Readerfit. Intra-assay variation; CV 5.4–6.99%, inter-assay variation; CV 4.34–9.96%. Progesterone concentrations are expressed in ng/ml.

## Results

### Development of a Modified Model of Mouse Menses

In the current study a mouse model of menstruation described by Brasted et al [15] was modified to include non-surgical induction of decidualisation and recovery of uterine tissues during a period of active shedding/repair which in our model spanned a period of 4 to 24 hours after removal of the progesterone pellet (progesterone withdrawal) (Figure 1). In pilot studies, during which uterine tissue was recovered 49 h after administration of oil via trans-uterine injection, the degree of decidualisation (increase in wet weight or presence of luminal decidual cell mass) in the strain of mice used in our laboratory was highly variable. In their original publication Finn and Pope [14] recorded a time-dependent increase in decidualisation therefore we changed our protocol to allow full decidualisation to occur (90 hours) before withdrawal of P4. Notably a study published after we had modified our protocol [26] also left the P4 implant in place until 4 days (∼96 hours) after stimulation with oil. An additional refinement of the protocol was the introduction of a small volume of oil via the vagina and cervix using a pipette tip designed for mouse IVF. We adopted the trans-vaginal route to avoid subjecting the steroid-stimulated uterine horn to the ‘insult’ of a trans-myometrial injection as the impact of this on tissue function was uncertain.

**Figure 2:**
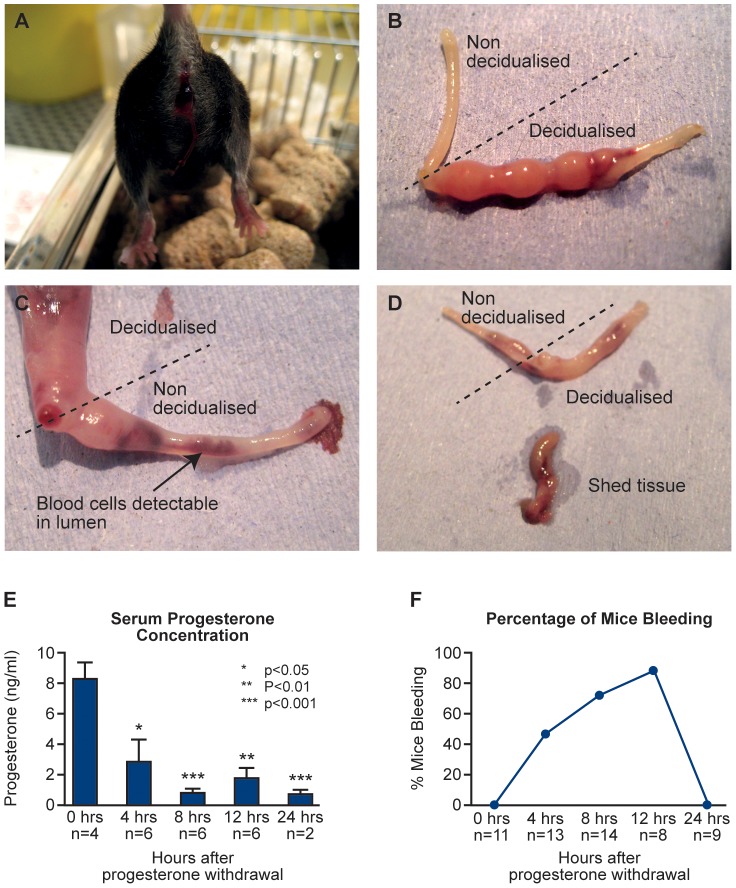
Gross morphology, bleeding and progesterone concentrations. A, Mouse at 12 hours after P4 withdrawal showing blood in vagina; B, The non decidualised control (left) and the decidualised horn (right) upon dissection (12 hours after withdrawal); C, Blood cells are detectable in lumen of the non decidualised horn following vaginal lavage (24 hours after withdrawal); D, Shed tissue expelled from cervix (24 hours after withdrawal), decidualised horn shows regression; E, Serum concentrations of progesterone (ng/ml). Statistical analysis was carried out by Student t test, comparing each time-point to the 0 hour time-point *p<0.05 **p<0.01 and ***p<0.001; F. Percentage of mice bleeding between 4 and 24 hours. This was calculated as a percentage of mice that were identified to be bleeding at each time-point of the total number of mice examined at each time-point.

Vaginal bleeding was observed as early as 4 hours after P4 withdrawal, was present in 87.5% of animals at 12 hours (Figure 2A). Removal of the uterus at different times after P4 withdrawal revealed evidence of intense unilateral decidualisation responses (Figure 2B) and time dependent-breakdown of the decidualised tissue (Figure 2C–D). Blood cells can be observed in the non-decidualised horn following vaginal lavage, where these cells are flushed out of the decidualised horn (Figure 2C). In line with expectations removal of the P4 pellet was associated with a rapid fall in serum concentrations of progesterone (Figure 2E) such that it was <50% at 4 hours (p<0.05). Overt bleeding had stopped by 24 hours (Figure 2F).

Histological evaluation of transverse tissue sections showed a decidual cell mass filling the lumen of the horn at time of P4 withdrawal consistent with a robust decidualisation response. As in women, variation in the breakdown and shedding of decidual tissue was observed; in all tissues examined, dissociation of the decidual mass from the underlying endometrium was associated with an apparent loss of tissue integrity (Figure S1). Shedding of the decidual mass resulted in portions of the stroma becoming denuded of overlying epithelium (Figure S2).

### Immunolocalisation of BrdU Suggested Proliferation of Epithelial Cells could Contribute to Restoration of an Intact Luminal Epithelium

**Figure 3:**
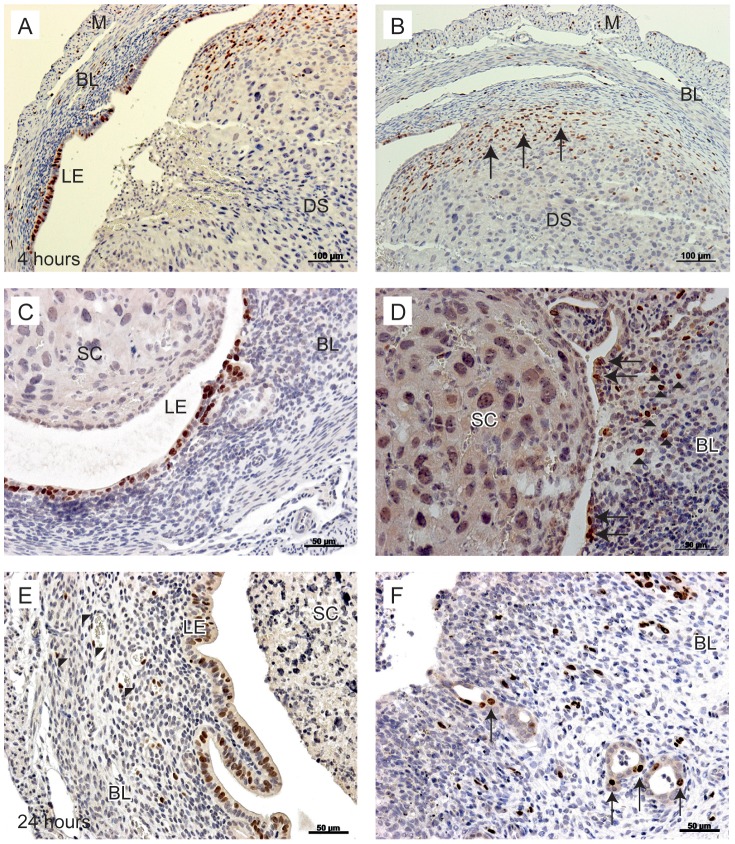
Proliferation of uterine cells between 4 and 24 hours after P4 withdrawal. To identify proliferating cells, animals were injected with BrdU 90 minutes prior to tissue recovery. A; Proliferating luminal epithelial cells detected in tissues 4 hours after progesterone withdrawal. B; In the same tissue, stromal cells in the basal layer are positive for BrdU (arrows). C; At 12 hours, luminal epithelial cells were positive for BrdU, no BrdU positive cells were identified in the shed cell mass. D; In the same tissue, stromal cells close to the luminal edge were positive for BrdU (arrowheads), new epithelial cells were identified lining the lumen in an area of tissue where the decidualised tissue had shed (arrows). E; At 24 hours after withdrawal, endothelial cells were positive for BrdU (arrowheads), the intact luminal epithelium was also positive for BrdU. F; In another sample at 24 hours, the stromal compartment was exposed to the lumen (arrowheads); note stromal cells positive for BrdU are interspersed throughout the basal layer and evidence of glandular epithelial cell proliferation was also detected (arrows). BL; Basal layer, LE; luminal epithelium, DS; decidualised stromal cells, M; myometrium, SC; shed cells. Scale bars are equal to 100 µm or 50 µm where indicated.

Cells that were actively proliferating at the time of tissue recovery were detected by incorporation of BrdU into cell nuclei during the 90 minutes prior to cull in uterine tissue sections at all time points. At the time of progesterone withdrawal (0 hours) stromal cells in the basal compartment and a few of the luminal epithelial cells were proliferating (not shown). At 4 (Figure 3A–B) and 12 (Figure 3C–D) hours many proliferating cells were present in both the basal stroma and epithelial layer lining the lumen. Proliferation of epithelial cells continued at the luminal surface at 24 hours and was also detectable in a few epithelial cells lining the basal glands (Figure 3E–F).

### Immunohistochemistry and Quantitative Analysis of Genes Associated with Epithelial and Stromal Cell Phenotype Provided Evidence for Re-epithelialisation by Epithelial Cells at the Luminal Surface

**Figure 4:**
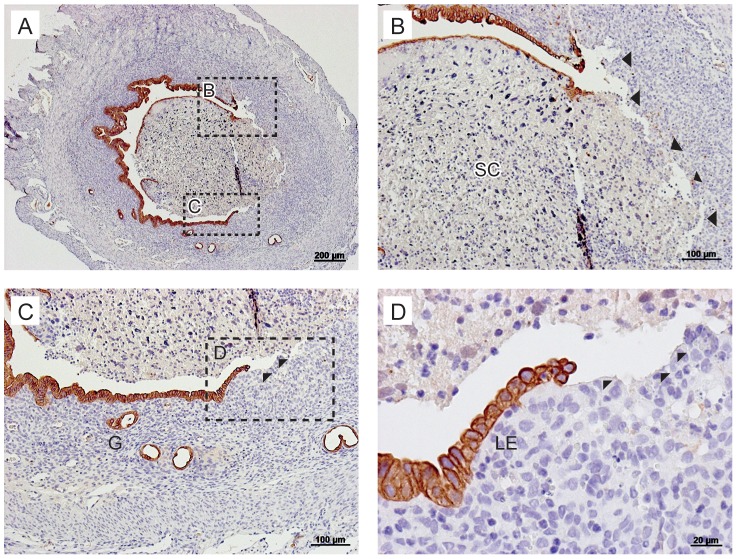
Immunostaining for pan-cytokeratin illustrates re-epithelialisation of the endometrium consistent with cell migration. Pan-cytokeratin, used as a marker for epithelial cells, was observed in the luminal epithelium and the glandular epithelium. A; The shed decidualised cell mass was observed to be detaching from the underlying stromal cell compartment at 24 hours after progesterone withdrawal. B; The denuded, underlying stroma, as indicated by the arrowheads, next to a region of luminal epithelial cells. C; The luminal epithelium next to an area of denuded basal stroma (arrowheads). D; Round epithelial cells appear to be “rolling” along an area of the denuded basal stroma. LE; luminal epithelium, G: glandular epithelium, SC; shed cells. Scale bars are equal to 200 µm, 100 µm or 20 µm where indicated.

In samples recovered at all time-points, where the shed decidual mass had detached from the underlying basal layer, areas of denuded stroma were documented immediately adjacent to regions where luminal epithelial cells remained intact (Figure S1). Epithelial cells in regions of the lumen and cells lining the basal glands are strongly immunopositive for cytokeratin (Figure 4A–C). In Figure 4D, the luminal epithelium adjacent to an area of denuded endometrium is shown, as depicted by the arrowheads. Adjacent to this region is what appears to be a ‘leading edge’ of rounded luminal epithelial cells that appear to be “rolling” across an area of the denuded stroma.

**Figure 5:**
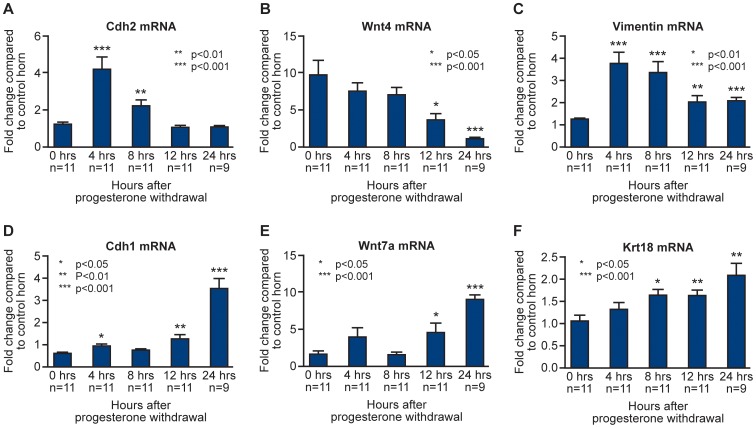
Dynamic changes in concentration of mRNAs specific to stromal and epithelial cell compartments. Comparison between concentrations of mRNAs encoded by genes typically expressed in stromal (*Cdh2, Wnt4*, vimentin) and epithelial (*Cdh1, Wnt7a, Krt8*) cells at 0 hours (full decidualisation) and following P4 withdrawal 4, 8, 12 and 24 hours prior to tissue recovery. Statistical analysis was performed by Student t test, comparing each time-point to the 0 hour time-point: *p<0.05, **p<0.01, ***p<0.001.

To complement the histological evaluations depicted in Figure 4, analysis of mRNA concentrations for known markers of cellular phenotype were investigated by qRTPCR. Dynamic changes in gene expression and an apparent reciprocal relationship between mRNAs expressed by known stromal cell markers (*Cdh2, Wnt4*, vimentin) and epithelial cell markers (*Cdh1, Wnt7a, Krt18*) were documented (Figure 5).

For example, significant increases in mRNA concentrations for definitive stromal cell markers N cadherin (*Cdh2*) and vimentin were detected at 4 and 8 hours after progesterone withdrawal (Figure 5A and C, p<0.01, p<0.001) consistent with relative increases in stromal cells positive for BrdU at this time (Figure 3A–D). Concentrations of *Wnt4* mRNAs (known to be involved in endometrial stromal cell development *in utero*) were maintained until 8 hours (Figure 5B) before decreasing at 12 and 24 hours, similar decreases were observed for *Cdh2* and vimentin at this time (Figure 5A and C). Consistent with epithelial cell proliferation (Figure 3 A, C, E) and the pattern of cytokeratin staining reported above (Figure 4) there was a progressive, significant, increase in the total concentration of *Krt18* mRNA from 8 to 24 hours (Figure 5F). Notably, at 12 and 24 hours *Cdh1* (E-Cadherin) a key marker of endometrial cell identity, was significantly increased compared with the 0 hour time-point (Figure 5D, p<0.001). mRNA concentrations for *Wnt7a,* known to play a key role in endometrial epithelial cell formation during embryonic development, were also significantly increased at 12 and 24 hours (Figure 5E).

### Immunohistochemistry Suggested Some Epithelial Cells Originated in the Stromal Compartment

**Figure 6:**
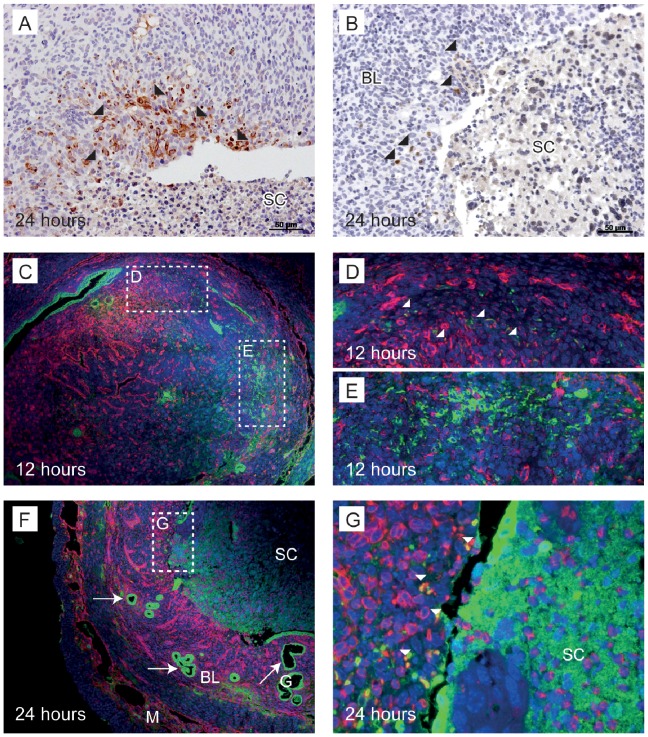
Cytokeratin positive cells were identified lining the luminal epithelium and glands as well as in the stroma with the latter appearing to be in transition between stromal and epithelial cell identity. A; A subset of stromal cells adjacent to the luminal surface were immunopositive for pan-cytokeratin at 24 hours after progesterone withdrawal (arrowheads). B; In another sample, positive pan-cytokeratin stromal cells (arrowheads) were detected adjacent to the luminal surface of a denuded area of endometrium. C; Immunofluorescence for epithelial cells stained for cytokeratin (green) and mesenchymal cells stained for vimentin (red) in the mouse endometrium. 12 hours after progesterone withdrawal, vimentin positive decidualised cells were observed, budding into the lumen. D; Vimentin and cytokeratin positive cells were observed in the stroma, close to the myometrium (arrowheads). E; stromal cells were positive for cytokeratin. F and G; 24 hours after progesterone withdrawal an area of shed endometrium is observed. Co-localisation of vimentin and cytokeratin (white arrowheads) was detected close to the surface of the underlying stroma. BL; basal layer, G; glandular epithelium, SC; shed cells, M; myometrium. Scale bars are equal to 50 µm where indicated.

Studies were undertaken to immunolocalise cytokeratin and vimentin as a complement to studies measuring their mRNAs in tissue homogenates (Figure 6, Figure S3). At all time-points, cells with intense immunostaining for cytokeratin were identified lining glands within the basal compartment of the endometrium (Figure S3B and C and Figure 6F, arrows). In samples recovered at 24 hours after P4 withdrawal cytokeratin-immunopositive cells were present within the stromal compartment but only in regions denuded of luminal epithelium (Figure 6A, B, depicted by arrowheads).

Dual immunofluorescence co-staining for vimentin and cytokeratin identified cells that were immunopositive for both vimentin and cytokeratin indicating that these cells had stromal and epithelial properties (Figure 6C–G, yellow, dual-labelled cells indicated by white arrowheads). At 12 hours, a few dual-labelled cells were detected in the stroma adjacent to the myometrium (Figure 6D) and cytokeratin positive cells (green) were present in the stroma (Figure 6E). At 24 hours, cells co-staining for vimentin and cytokeratin were identified in the stromal compartment adjacent to the lumen (Figure 6G, white arrowheads). These findings appear consistent with some stromal cells adopting a transition/intermediate phenotype prior to differentiation into epithelial cells.

### Array Analysis and Quantitative RTPCR Revealed Changes in Expression of Genes Associated with MET

Analysis of gene expression changes using a focused PCR array revealed a greater proportion of genes were up-regulated than down-regulated at both 8 and 24 hours when compared with 0 hours. Of the 21 changes in gene expression detected by comparing the 8 and 24 hour time points, 12 were up-regulated whereas 9 were down-regulated (Table 1). Those genes which displayed significant changes in gene expression are outlined in Tables S3 and S4. Consistent with results of qRTPCR analysis, expression of the cytokeratin (*Krt7*) and E-Cadherin (*Cdh1*) in the array were both markedly increased in samples recovered at 24 hours (Table S4). Other striking changes in gene expression at 24 hours (Table S4) would all be consistent with re-establishment of a mature, functional epithelial compartment associated with restoration of junctional integrity: cell adhesion molecules *Dsc2* (desmocollin 2, 4 fold increase) and *Spp1* (osteopontin, 23 fold increase) and the intracellular junction protein *Dsp* (desmoplakin, 9 fold increase).

**Table 1:** Number of gene changes in MET PCR array.

Time-point	No. Genes Up-regulated	No. Genes Down-regulated
8 hours (compared to 0 hours)	20	4
24 hours (compared to 0 hours)	43	5
24 hours (compared to 8 hours)	12	9

A number of changes detected on the array mirrored those reported in human tissue at time of menses, including a striking up-regulation in expression of *Mmp3* (a 3 fold increase at 8 hours, and a 61 fold increase at 24 hours); *Mmp9* was also significantly up-regulated between 8 and 24 hours (11 fold increase, not shown).

**Figure 7:**
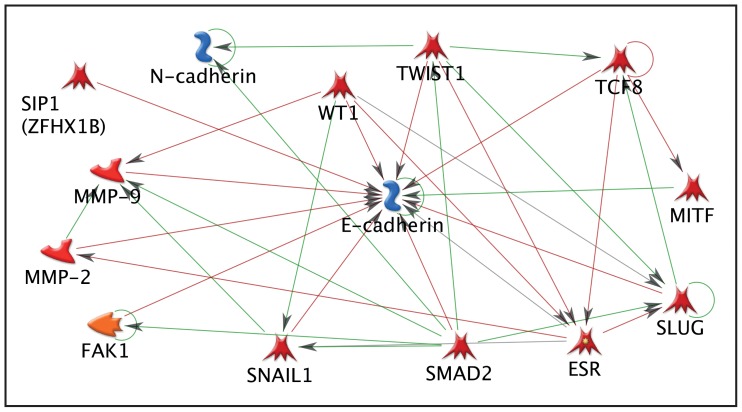
Metacore analysis of genes detected in mouse uterus that were associated with regulation of E cadherin and N cadherin. To filter data, the full gene array list was input into Metacore™ software and any gene that was found to have no known interaction with E and N cadherin was excluded. Arrows indicate direct effects on other genes in the pathway. Green arrows indicate activation, whereas red arrows show inhibitory action.

To complement and extend the data obtained using the PCR array, bioinformatic analysis was conducted using Metacore software (Figure 7). Analysis of genes identified as up-stream regulators of *Cdh1* and *Cdh2*, both of which changed in a dynamic way in our uterine tissue, highlighted both an association with MMPs as well as with *Smad2* which we have previously identified as involved in TGFß signaling in human endometrial stromal cells [27]. Six other genes were identified for further study, these were; WT1 (not represented on the array), Snail (*Snai1*) and Slug (*Snai2*) as all three have been identified as key regulators of transitions between the mesenchymal and epithelial states [reviewed in [28], Smuc (*Snai3*), *Twist* and *Mmp3*.

**Figure 8:**
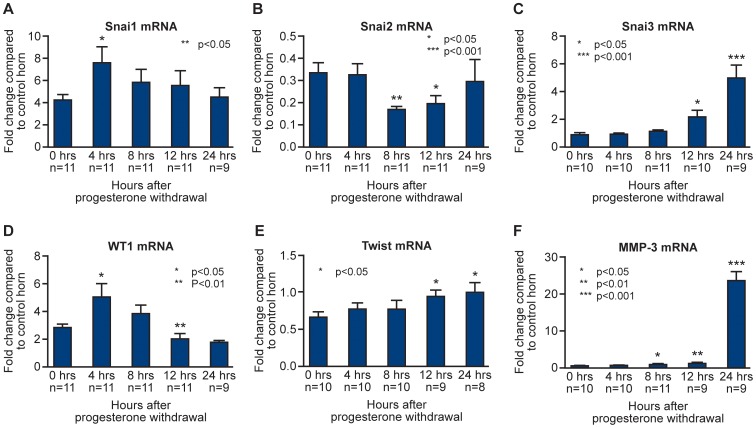
Endometrial remodelling was associated with dynamic changes in concentrations of mRNAs expressed in stromal and epithelial cell types as well as those encoded by genes implicated in MET. mRNA concentrations for candidate genes involved in mesenchymal to epithelial transition and tissue remodelling; *Snai1* (Snail), *Snai2* (Slug), *Snai3* (Smuc), *Wt1*, *Twist* and *Mmp3* following progesterone withdrawal. mRNA expression for the decidualised horn (black bars) was normalised against the control 0 hour horn. Statistical analysis was performed by Student t test, comparing each time-point to the decidualised 0 hour time-point, *p<0.05, **p<0.01, ***p<0.001.

QRTPCR analysis revealed a transient and significant increase in expression of *Wt1* and *Snai1* at 4 hours (P<0.05, Figure 8 D and A respectively) and a significant decrease in *Snai2* (Slug) at 8 and 12 hours (Figure 8B) which is in agreement with the results obtained using the PCR array which detected a 2 fold decrease in the 8 hour sample (4 and 12 hours not examined). *Snai3*, (Figure 8C) not previously studied in the endometrium, was significantly increased at 12 and 24 hours, consistent with results obtained using the PCR array (15 fold increase at 24 hours). A gradual increase in *Twist* was detected (Figure 8E), with a significant increase at 12 and 24 hours. Consistent with findings in the array, *Mmp3* was significantly up-regulated at 8, 12 and 24 hours (Figure 8F), with the highest expression at 24 hours (consistent with the 61 fold increase obtained in the array).

**Figure 9:**
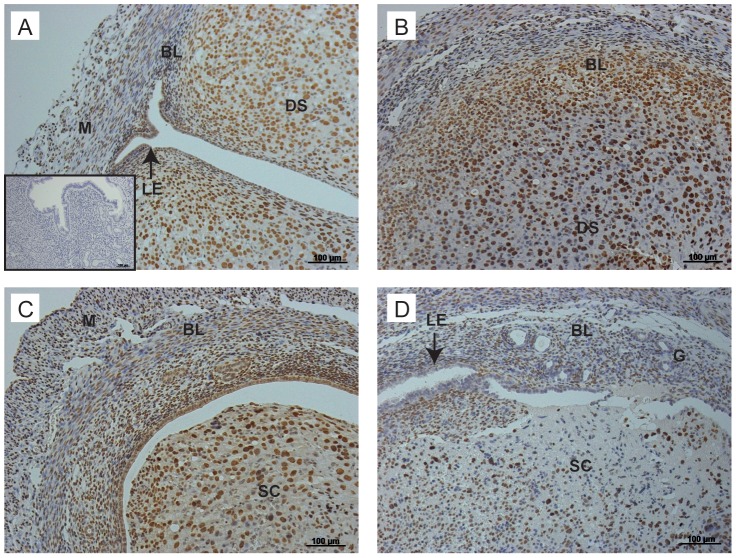
Immunostaining for WT1 illustrates dynamic changes in cellular localisation during breakdown and repair. A; Immunopoistive staining for WT1 was detected in decidualised stromal cells, stromal cells of the basal layer and the luminal epithelium at the time of progesterone withdrawal. B; At 4 hours, strong immunostaining was maintained in the basal and decidual layers of the tissue. C; By 8 hours, strong immunopositive staining was localised to areas close to the luminal epithelium. D; By 12 hours, fewer immunopositive cells were observed, these were limited to the stroma, no immunostaining for WT1 was detected in the luminal epithelium. LE; luminal epithelium, G: glandular epithelium, SC; shed cells, DS; decidualised stroma, M; myometrium. Inset; negative control. Scale bars are equal to 100 µm where indicated.

To complement the QRTPCR analysis, immunohistochemistry for WT1 was carried out to determine cellular localisation of the protein (Figure 9). At the time of progesterone withdrawal (0 hours, Figure 9A) nuclei of the decidualised stromal cells, stromal cells in the basal layer and luminal epithelial cells were positive for WT1. Consistent with the changes in mRNA concentrations, WT1 expression was maintained at 4 hours (Figure 9B) and 8 hours (Figure 9C) however by 12 hours (Figure 9C) WT1 expression had decreased and was limited to the stromal cell compartment.

## Discussion

In women, extensive tissue remodelling occurs during menses with restoration of homeostasis requiring rapid and co-ordinated re-epithelialisation, stromal cell remodelling and endothelial cell proliferation. Studies on the regulation of endometrial repair have been informed by studies in mouse models using two different induced models of tissue shedding. The most widely used model is the one originally proposed by Finn and Pope [14] that was modified and widely utilised by the Salamonsen laboratory [15]–[17] and revised in the current paper. Notably, mirroring the situation in women where endometrial repair occurs at a time when both oestradiol and progesterone levels are low, termed a “steroid-depleted” environment, endometrial repair was reported to occur in the absence of oestrogen in a mouse model of endometrial breakdown [16]. A second mouse model relies on formation of a corpus luteum following mating with a vasectomised male, this induces a rise in progesterone and makes the uterus receptive to a decidualising stimulus; progesterone withdrawal can be induced by administration of an antiprogestin [29], [30]. Of these mouse models overt vaginal bleeding has only been detected in the current paper and in two recent studies by Rudolf, Roese and colleagues [26], [29] one of which used the pseudopregnant model [29]. A key feature of our study and that of Menning et al [26] was the induction of a substantial and sustained decidual response stimulated by P4 released by an implant left in place for 90–96 hours after introduction of oil into the uterine lumen. In our model, endometrial breakdown occurred within 4 hours of the withdrawal of progesterone, consistent with a 70% decrease in serum progesterone concentrations and evidence of blood in the vaginal smear. A direct comparison with the other models in which bleeding was recorded [26] is not possible because tissue was not recovered until 24 hours after P4 withdrawal. In a recent paper, a critical period of progesterone withdrawal was reported to precede endometrial breakdown [31] and the authors reported that replacement of progesterone after 12–16 hours was sufficient to reverse the effects of progesterone withdrawal. However, the authors do not report any evidence of endometrial bleeding until 12 hours after the withdrawal of progesterone, despite a reported drop in progesterone concentrations from ∼128 ng/ml to ∼14 ng/ml in the first 8 hours [31]. In our model, circulating progesterone concentrations are maximal prior to progesterone pellet removal at 8 ng/ml, bleeding is recorded at 4 hours when progesterone concentrations are ∼3 ng/ml. Therefore it is likely that it is not the drop in progesterone that triggers breakdown but a minimal progesterone concentration threshold, which once the level has dropped below it, it cannot be recovered.

Results obtained with the current model mimic features of human menstruation including disturbances in expression of epithelial and junctional proteins such as desmoplakin [32] and significant increases in expression of MMPs [33]. Studies in mice with a conditional knockout of *Cdh1* have demonstrated an essential role for the gene in development of the uterus with knockout mice having abnormal epithelial development and reduced expression of cytokeratin 8 and occludin [34]; a link between expression of these key epithelial cell protein components was also observed in the current study with expression of *Cdh1*, cytokeratins (*Krt*19, 7) and occludin (*Ocln*) all being up-regulated in our 24 hour samples.

Detailed immunohistochemical evaluation provided evidence that stromal cells adjacent to areas denuded of epithelium were in transition between having a mesenchymal (vimentin positive) cell fate and an epithelial one, indicative of MET. Notably, although we have no data from normal menstruating human endometrial tissue, transitions between a epithelial and mesenchymal state plays a fundamental role in formation of tissue systems with expression of WT1 playing a pivotal role in regulating cell fate [35].

Huang et al [36] used genetic fate mapping to examine uterine regeneration and remodeling following parturition. They found evidence that stromal and epithelial compartments maintain separate fates during normal oestrous cycles, but in restoration of tissue integrity following parturition a subset of stromal cells differentiate to become incorporated into luminal and glandular epithelium. In our model of induced menses we first noted stromal cells that were in a transitional state within the basal compartment at 12 hours after progesterone withdrawal with a more robust response at 24 hours, the latter being in position to complement the restoration of an intact epithelial layer by migrating/proliferating epithelial cells. A study recently published by Patterson et al [30] examined uterine tissue recovered following parturition, as well as a menses model based on induced pseudopregnancy and progesterone withdrawal by ovariectomy. Their results using reporter lines confirmed those of Huang et al and they also found a subset of stromal cells double stained for vimentin and cytokeratin located close to the myometrial epithelial border 24 hours after ovariectomy. In this study we examined expression of genes known to regulate the process of MET. These extend our previous studies on the role of TGFß1, a protein documented as a regulator of EMT in endometrial cancer [37], in regulation of endometrial stromal cells. In a previous study we found evidence for Smad2 dependent suppression of expression of progesterone receptor [27]. In the current study we documented changes in expression of WT1 as well as Snai1, 2 and 3. Changes in WT1 and Snai1 were both transient occurring in the immediate phase of tissue shedding. We also detected a robust increase in expression of Snai3 (5 fold at 8 hours, 15 fold at 24 hours). Mice with knockout of Snai3 are viable and fertile but double knockout of Snai2/3 resulted in marked depletion of bone-marrow derived cells [38] raising the possibility that Snai3 may play a previously unrecognised role in regulation of bone-marrow derived cells in the uterus [39]. We also speculate that reduced expression of Snai2 (Slug) at 8 and 12 hours might relieve repression of the *Cdh1* gene favouring epithelial cell fate. As the decidualised functional layer sheds, it is possible that it secretes factors that initiate repair. In our mouse model, progressive shedding of the decidualised functional stroma was observed (8 hours after progesterone withdrawal); in addition to this, residual luminal epithelial cells were observed to be “moving” towards sites of newly exposed endometrium. We postulate that as the endometrium detaches from the underlying basal stroma, it secretes factors that promote repair. A study by Gaide-Chevronnay et al on human tissues, supports a role for the degenerating endometrium supporting its own repair [11]. Evidence includes an increase in leukocyte chemokines, extracellular matrix (ECM) proteins and enzymes involved in prostaglandin synthesis were detected in the functional layer of menstrual phase tissue. Furthermore, a scanning electron microscopy study by Garry et al observed that immediately after shedding, a fibrinous matrix appears to “seal” the endometrium prior to re-epithelialisation [3]. In support of this a previous mouse model of breakdown has shown that extracellular matrix proteins were increased at the time of repair and were localised to the luminal edge of the uterine horn [17].

A role for the degenerating functional stroma in endometrial repair has not been widely studied. Although, it is tempting to speculate that it may play a role in the pathology of endometrial disorders. For example, women with heavy menstrual bleeding may have a dysfunctional stroma that does not secrete repair factors, resulting in delayed repair and a longer bleed.

## Conclusions

The current study has used an updated mouse model to investigate the mechanisms that contribute to restoration of tissue integrity following shedding of the uterine lining. These studies have revealed a potentially important role for MET in the complex cellular dynamics that underpin rapid healing of the endometrial lining each cycle and have implications for management of women suffering from disturbances in endometrial function, including heavy bleeding, and development of new non-surgical therapies for these conditions.

## Supporting Information

Figure S1 **Loss of endometrial integrity during endometrial breakdown.** Haematoxylin and eosin staining of tissues collected at 8 and 12 hours after progesterone withdrawal. A; The functional decidualised stroma detaches basal layer resulting in exposed regions of underlying stroma (arrows). B; At 12 hours, the shed cell mass disaggregates with the underlying stroma (arrows). SC; shed cells. Scale bars are equal to 50 µm where indicated.
(TIF)

Figure S2 **Shedding results in a denuded stromal cell compartment.** Haematoxylin and eosin staining of tissues collected at 24 hours after progesterone withdrawal. A and B; shedding of the functional stroma (SC) results in areas of denuded basal stroma, with no evidence of luminal epithelial cells (arrowheads). SC; shed cells. Scale bars are equal to 50 µm where indicated.
(TIF)

Figure S3 **Epithelial cell dynamics during endometrial breakdown.** Pan-cytokeratin, used as a marker for epithelial cells, was observed in the luminal epithelium and the glandular epithelium at 0 and 4 hours after progesterone withdrawal. A; At 0 hours, the luminal epithelium is immunopositive for pan-cytokeratin. A cluster of cells in the decidualised stroma are also positive (arrow). B; in the same tissue, weak immunostaining for cytokeratin was detected in the decidualised stroma. C; glands in the basal stroma are immunopositive for cytokeratin at 4 hours after progesterone withdrawal. D; in the same tissue the leading edge of the decidualised stroma, that is beginning to breakdown, is immunopositive (arrowheads). LE; luminal epithelium, DS; decidualised stroma, BL; basal layer, G; glandular epithelium. Scale bars are equal to 100 µm where indicated.
(TIF)

Table S1 **Details of Antibodies.**
(DOCX)

Table S2 **Primer sequences, accession numbers and UPL probe numbers used for genes of interest.**
(DOCX)

Table S3 **Significant changes in gene expression 8 hours after progesterone withdrawal, as displayed by up- or down- fold regulation when compared against the 0 hour group, n = 6.**
(DOCX)

Table S4 **Significant changes in gene expression 24 hours after progesterone withdrawal, as displayed by up- or down- fold regulation when compared against the 0 hour group, n = 6.**
(DOCX)
