# Supplementary figures and images for: Evidence from a Mouse Model That Epithelial Cell Migration and Mesenchymal-Epithelial Transition Contribute to Rapid Restoration of Uterine Tissue Integrity during Menstruation
Source: PLoS One. 2014 Jan 22;9(1):e86378. doi: 10.1371/journal.pone.0086378 (PMC3899239; doi:10.1371/journal.pone.0086378)

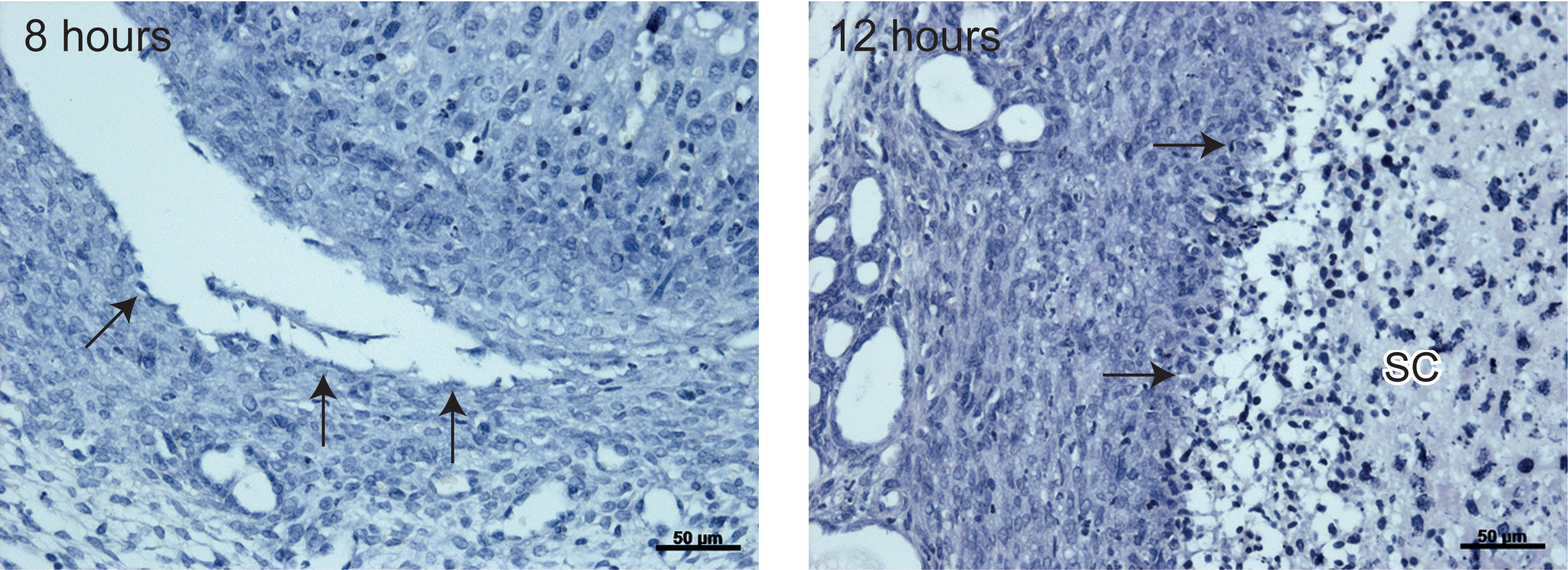

Supplement: Figure S1 — Loss of endometrial integrity during endometrial breakdown. Haematoxylin and eosin staining of tissues collected at 8 and 12 hours after progesterone withdrawal. A; The functional decidualised stroma detaches basal layer resulting in exposed regions of underlying stroma (arrows). B; At 12 hours, the shed cell mass disaggregates with the underlying stroma (arrows). SC; shed cells. Scale bars are equal to 50 µm where indicated. (TIF) [file pone.0086378.s001.tif]

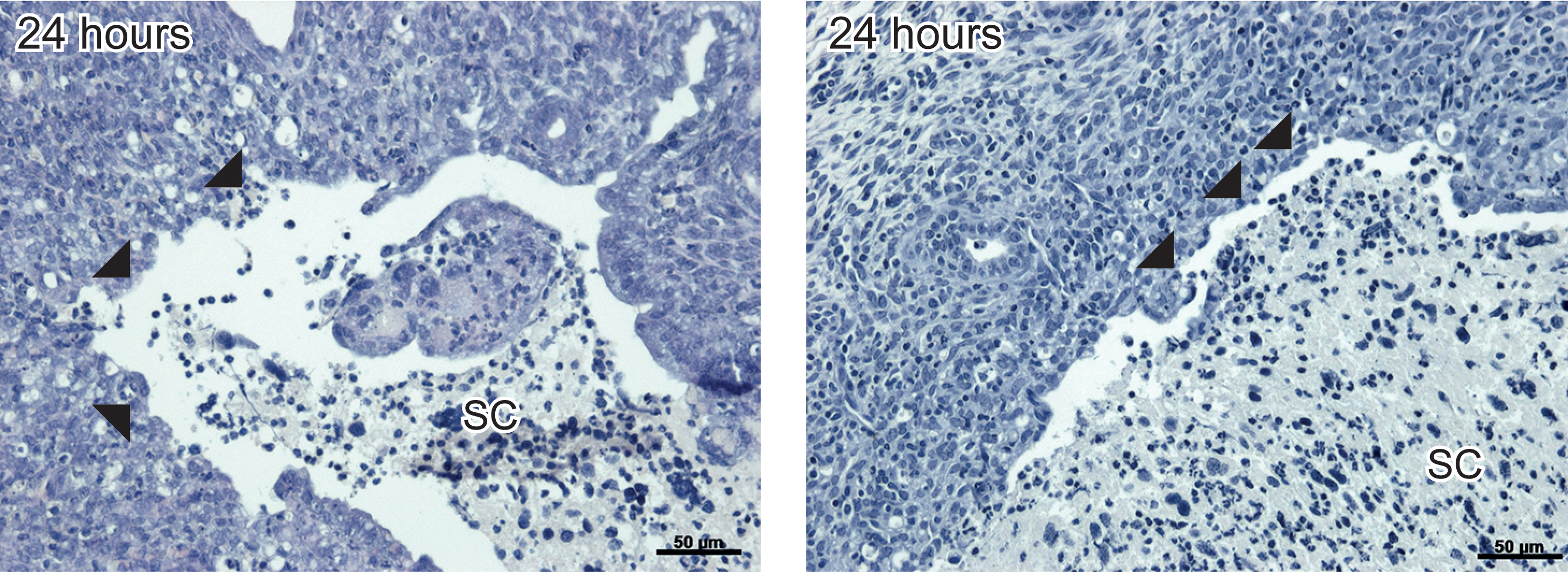

Supplement: Figure S2 — Shedding results in a denuded stromal cell compartment. Haematoxylin and eosin staining of tissues collected at 24 hours after progesterone withdrawal. A and B; shedding of the functional stroma (SC) results in areas of denuded basal stroma, with no evidence of luminal epithelial cells (arrowheads). SC; shed cells. Scale bars are equal to 50 µm where indicated. (TIF) [file pone.0086378.s002.tif]

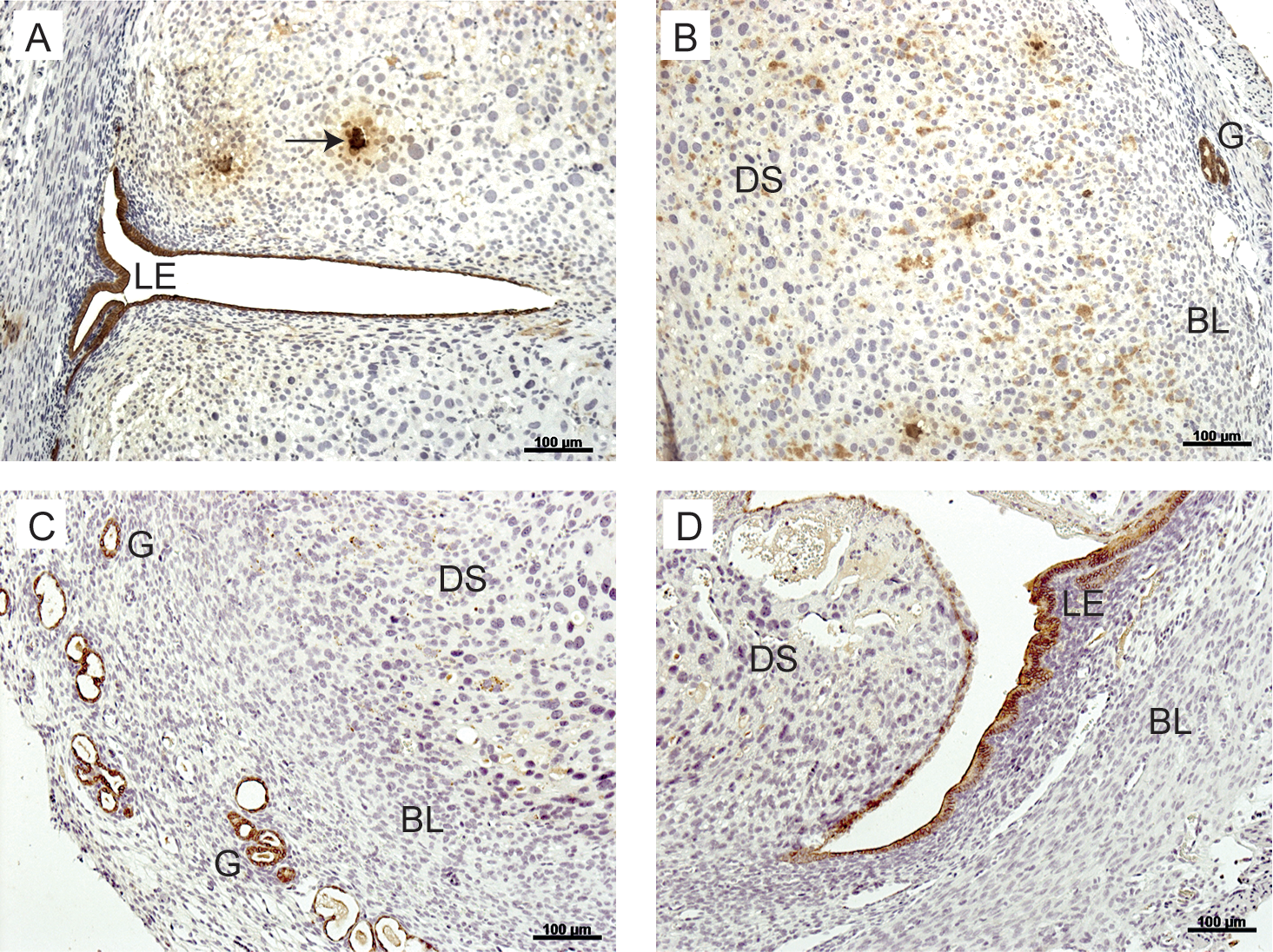

Supplement: Figure S3 — Epithelial cell dynamics during endometrial breakdown. Pan-cytokeratin, used as a marker for epithelial cells, was observed in the luminal epithelium and the glandular epithelium at 0 and 4 hours after progesterone withdrawal. A; At 0 hours, the luminal epithelium is immunopositive for pan-cytokeratin. A cluster of cells in the decidualised stroma are also positive (arrow). B; in the same tissue, weak immunostaining for cytokeratin was detected in the decidualised stroma. C; glands in the basal stroma are immunopositive for cytokeratin at 4 hours after progesterone withdrawal. D; in the same tissue the leading edge of the decidualised stroma, that is beginning to breakdown, is immunopositive (arrowheads). LE; luminal epithelium, DS; decidualised stroma, BL; basal layer, G; glandular epithelium. Scale bars are equal to 100 µm where indicated. (TIF) [file pone.0086378.s003.tif]
