# Supplementary material for: Evidence from a Mouse Model That Epithelial Cell Migration and Mesenchymal-Epithelial Transition Contribute to Rapid Restoration of Uterine Tissue Integrity during Menstruation
Source: PLoS One. 2014 Jan 22;9(1):e86378. doi: 10.1371/journal.pone.0086378 (PMC3899239; doi:10.1371/journal.pone.0086378)
Supplement: Table S1 — Details of Antibodies. (DOCX) [file pone.0086378.s004.docx]

*Primary Antibodies*

| **Name** | **Supplier** | **Cat No** | **Dilution IHC** | **Dilution IF** | **Serum Block** |
| --- | --- | --- | --- | --- | --- |
| BrdU | Fitzgerald | 20-BS17 |  | 1/4000 | Rabbit |
| Pan cytokeratin | Sigma | C2562 | 1/2000 | 1/4000 | Goat |
| Vimentin | Cell Signalling Tech | #5741 |  | 1/600 | Goat |
| WT1 | Santa Cruz | sc-192 | 1/1000 |  | Goat |

*Secondary Antibodies*

| **Name** | **Supplier** | **Cat No** |
| --- | --- | --- |
| Rabbit anti sheep peroxidase | AutogenBioclear | ABN001HRP |
| Goat anti rabbit peroxidase | Vector | PI 1000 |
| Goat anti mouse biotinylated | Vector | BA 9200 |
| Goat anti rabbit biotinylated | Vector | BA1000 |
| Goat anti mouse peroxidase | Abcam | Ab6823 |
