# Supplementary material for: Evidence from a Mouse Model That Epithelial Cell Migration and Mesenchymal-Epithelial Transition Contribute to Rapid Restoration of Uterine Tissue Integrity during Menstruation
Source: PLoS One. 2014 Jan 22;9(1):e86378. doi: 10.1371/journal.pone.0086378 (PMC3899239; doi:10.1371/journal.pone.0086378)
Supplement: Table S2 — Primer sequences, accession numbers and UPL probe numbers used for genes of interest. (DOCX) [file pone.0086378.s005.docx]

| Gene name | Accession Number | Forward Primer Sequence | Reverse Primer Sequence | UPL Probe Number |
| --- | --- | --- | --- | --- |
| Mus musculus cadherin 1 *(Cdh1)* | NM_009864.2 | atcctcgccctgctgatt | accaccgttctcctccgta | 18 |
| Mus musculus cadherin 2 *(Cdh2)* | NM_007664.4 | cctccatgtgccggatag | caccagaagcctccacagac | 74 |
| Mus musculus wingless-related MMTV integration site 4 *(Wnt4)* | NM_009523 | ctggactccctccctgtctt | atgcccttgtcactgcaaa | 62 |
| Mus musculus wingless-related MMTV integration site 7a *(Wnt7a)* | NM_009527.3 | cgctcatgaacttacacaataacg | acaggagcctgacacaccat | 78 |
| Mus musculus Vimentin *(Vim)* | NM_011701.4 | tgcgccagcagtatgaaa | gcctcagagaggtcagcaaa | 79 |
| Mus musculus keratin 18 *(Krt18)* | NM_010664.2 | agatgacaccaacatcacaagg | tccagaccttggacttcctc | 78 |
| Mus musculus Wilms tumor 1 homolog *(Wt1)* | NM_144783.2 | cagatgaacctaggagctaccttaaa | tgcccttctgtccatttca | 3 |
| Mus musculus snail homolog 1 (Drosophila) (*Snai1)* | NM_011427.2 | gtctgcacgacctgtggaa | caggagaatggcttctcacc | 71 |
| Mus musculus snail homolog 2 (Drosophila) *(Snai2)* | NM_011415.2 | tgcaagatctgtggcaagg | cagtgagggcaagagaaagg | 71 |
| Mus musculus snail homolog 3 (Drosophila) (Snai3) | NM_013914.2 | gtccccaactacgggaaact | gggatcctgccaactcct | 15 |
| Mus musculus twist homolog 1 (Drosophila) (Twist1) | NM_011658.2 | agctacgccttctccgtct | tccttctctggaaacaatgaca | 58 |
| Mus musculus matrix metallopeptidase 3 (Mmp3) | NM_010809.1 | ttgttctttgatgcagtcagc | gatttgcgccaaaagtgc | 7 |
