# Supplementary material for: Evidence from a Mouse Model That Epithelial Cell Migration and Mesenchymal-Epithelial Transition Contribute to Rapid Restoration of Uterine Tissue Integrity during Menstruation
Source: PLoS One. 2014 Jan 22;9(1):e86378. doi: 10.1371/journal.pone.0086378 (PMC3899239; doi:10.1371/journal.pone.0086378)
Supplement: Table S3 — Significant changes in gene expression 8 hours after progesterone withdrawal, as displayed by up- or down- fold regulation when compared against the 0 hour group, n = 6. (DOCX) [file pone.0086378.s006.docx]

| **Gene Name** | **p value** | **Fold Regulation** |
| --- | --- | --- |
| *Col5a2* | 0.014999 | 1.3733 |
| *Fzd7* | 0.004084 | 2.112 |
| *Gng11* | 0.027026 | -2.0012 |
| *Igfbp4* | 0.007334 | 1.8312 |
| *Il1rn* | 0.000873 | 2.0872 |
| *Ilk* | 0.027785 | 1.4167 |
| *Itgb1* | 0.001503 | 1.4681 |
| *Krt19* | 0.000383 | 1.9973 |
| *Krt7* | 0.001239 | 4.8209 |
| *Mmp2* | 0.008265 | 2.0402 |
| *Mmp3* | 0.013284 | 3.0056 |
| *Mtap1b* | 0.021019 | 1.7783 |
| *Pdgfrb* | 0.030445 | 2.1571 |
| *Plek2* | 0.001445 | 2.1191 |
| *Rgs2* | 0.001027 | -2.7773 |
| *Snai2* | 0.018714 | -2.1138 |
| *Snai3* | 0.006822 | 5.6312 |
| *Spp1* | 0.001467 | 4.3785 |
| *Stat3* | 0.001286 | 1.5821 |
| *Steap1* | 0.000673 | 2.3442 |
| *Tfpi2* | 0.008769 | -1.8252 |
| *Timp1* | 0.015419 | 1.6154 |
| *Tmeff1* | 0.000078 | 2.3973 |
| *Wnt11* | 0.000277 | 3.4224 |
