# Supplementary material for: Evidence from a Mouse Model That Epithelial Cell Migration and Mesenchymal-Epithelial Transition Contribute to Rapid Restoration of Uterine Tissue Integrity during Menstruation
Source: PLoS One. 2014 Jan 22;9(1):e86378. doi: 10.1371/journal.pone.0086378 (PMC3899239; doi:10.1371/journal.pone.0086378)
Supplement: Table S4 — Significant changes in gene expression 24 hours after progesterone withdrawal, as displayed by up- or down- fold regulation when compared against the 0 hour group, n = 6. (DOCX) [file pone.0086378.s007.docx]

| **Gene Name** | **p value** | **Fold Regulation** |
| --- | --- | --- |
| *Akt1* | 0.004507 | 1.6241 |
| *Bmp1* | 0.001376 | 1.7233 |
| *Bmp7* | 0.04878 | 1.8035 |
| *Cald1* | 0.006957 | 2.2812 |
| *Camk2n1* | 0.04425 | 4.8329 |
| *Cav2* | 0.03615 | 1.7465 |
| *Cdh1* | 0 | 5.1764 |
| *Cdh2* | 0.046312 | 1.4957 |
| *Dsc2* | 0.000383 | 4.734 |
| *Dsp* | 0.000257 | 9.5533 |
| *Esr1* | 0.032215 | 1.5223 |
| *Fn1* | 0.007739 | 3.0108 |
| *Fzd7* | 0.011578 | 2.2833 |
| *Gng11* | 0.016563 | -2.7033 |
| *Gsc* | 0.039591 | 2.8029 |
| *Gsk3b* | 0.046881 | 1.4052 |
| *Ilk* | 0.004121 | 1.8766 |
| *Itgb1* | 0.006007 | 1.6552 |
| *Jag1* | 0.002575 | 3.4856 |
| *Krt19* | 0.000053 | 7.5293 |
| *Krt7* | 0.000018 | 28.178 |
| *Mitf* | 0.001484 | -2.1751 |
| *Mmp2* | 0.00544 | 2.7303 |
| *Mmp3* | 0.001871 | 61.2906 |
| *Msn* | 0.000728 | 1.8437 |
| *Mtap1b* | 0.000492 | 2.1494 |
| *Notch1* | 0.031693 | 1.6165 |
| *Ocln* | 0.005327 | 11.2243 |
| *Pdgfrb* | 0.000015 | 4.5738 |
| *Plek2* | 0.044301 | 1.6349 |
| *Ptp4a1* | 0.002475 | 1.8968 |
| *Rgs2* | 0.001014 | -4.2015 |
| *Smad2* | 0.005844 | 1.3823 |
| *Snai3* | 0.011681 | 15.8243 |
| *Spp1* | 0.000141 | 23.2021 |
| *Stat3* | 0.00232 | 1.8157 |
| *Steap1* | 0.000283 | 4.6399 |
| *Tcf4* | 0.001468 | 1.4002 |
| *Tfpi2* | 0.002632 | -2.1709 |
| *Tgfb1* | 0.004964 | 1.783 |
| *Tgfb2* | 0.004658 | -1.497 |
| *Tmeff1* | 0.000001 | 4.1575 |
| *Tmem132a* | 0.00069 | 2.8066 |
| *Tspan13* | 0.00039 | 3.2687 |
| *Twist1* | 0.00125 | 3.294 |
| *Vim* | 0.001135 | 1.7446 |
| *Vps13a* | 0.035245 | 1.5401 |
| *Zeb1* | 0.000009 | 2.4496 |
